# Management of HER2-positive and microsatellite instability-high advanced gastric cancer: a case report

**DOI:** 10.1007/s13691-024-00707-0

**Published:** 2024-08-02

**Authors:** Taichi Tamura, Yusuke Kanemasa, Shohei Nakamura, Toshihiro Okuya, Yu Yagi, Shinichiro Matsuda, Mitsutaka Murata, Kazuya Endo, Kentaro Hara, Hiroko Okinaga, Shin-ichiro Horiguchi, Yasuji Seyama, Haruhiko Cho, Tatsu Shimoyama

**Affiliations:** 1 Department of Medical Oncology, Tokyo Metropolitan Cancer and Infectious Diseases Center, Komagome Hospital, 3-18-22 Honkomagome, Bunkyo-ku, Tokyo, 113-8677 Japan; 2 Department of Gastric Surgery, Tokyo Metropolitan Cancer and Infectious Diseases Center, Komagome Hospital, Tokyo, Japan; 3 Department of Hepato-Biliary-Pancreatic Surgery, Tokyo Metropolitan Cancer and Infectious Diseases Center, Komagome Hospital, Tokyo, Japan; 4 Department of Pathology, Tokyo Metropolitan Cancer and Infectious Diseases Center, Komagome Hospital, Tokyo, Japan

**Keywords:** Human epidermal growth factor receptor 2-positive, Microsatellite instability-high, Advanced gastric cancer, Trastuzumab

## Abstract

Chemotherapy for advanced gastric cancer has progressed significantly in the past few decades. Biomarker-specific drugs, including anti-human epidermal growth factor receptor 2 (HER2) drugs for HER2-positive patients and immune checkpoint inhibitors for those with microsatellite instability-high (MSI-H), have become common. However, patients who are positive for HER2 and have MSI-H are extremely rare, and there are no established treatments for these patients. We present the case of a 75-year-old, male patient with gastric cancer with lymph node metastases and liver infiltration. Biomarker analysis revealed HER2 3 + , loss of MLH1, and MSI-H. After three cycles of S-1, oxaliplatin, and trastuzumab, the primary tumor and metastases shrank markedly. He subsequently underwent gastrectomy and hepatectomy as conversion surgery, achieving a pathologically complete response. He has been recurrence-free for seven months postoperatively. The present case demonstrated the efficacy of trastuzumab-containing chemotherapy followed by conversion surgery in a patient with HER2-positive, MSI-H, advanced gastric cancer.

## Introduction

Human epidermal growth factor receptor 2 (HER2) is a transmembrane tyrosine kinase receptor that controls cell proliferation and cell cycle signaling pathways [1]. Approximately one-fifth of patients with gastric/gastroesophageal junction (GEJ) cancer have overexpression or amplification of HER2 [2], and anti-HER2 treatments have demonstrated remarkable improvement in these patients [3, 4]. Numerous studies have reported the efficacy of immune checkpoint inhibitors (ICIs) against advanced gastric cancer [5–8]. In particular, cancers with microsatellite instability-high (MSI-H) are a molecularly defined subset associated with a high probability of responsiveness to ICI therapy [9]. However, the frequency of MSI-H in patients with gastric cancer is as low as 6.7% [10]. Furthermore, HER2-positive and MSI-H gastric cancer is extremely rare, with a reported incidence of approximately 0.7% [11]. For this rare subset of patients with HER2-positive and MSI-H unresectable advanced gastric cancer, the optimal treatment strategy—whether chemotherapy plus trastuzumab or chemotherapy plus immune checkpoint inhibitors—has not yet been established.

Conversion surgery utilizes preoperative therapy to “convert” unresectable tumors into resectable ones before removing them via surgical resection. This approach has garnered attention as potential means of improving the survival outcomes of patients with advanced gastric cancer. Several studies have demonstrated the efficacy of conversion surgery in treating advanced gastric cancer [12–14]. However, its benefit for patients with HER2-positive, MSI-H gastric cancer remains unconfirmed.

Herein, we report a case of HER2-positive, MSI-H gastric cancer in which a significant response was achieved using a trastuzumab-based chemotherapy regimen, followed by conversion surgery.

## Case report

**Fig. 1:**
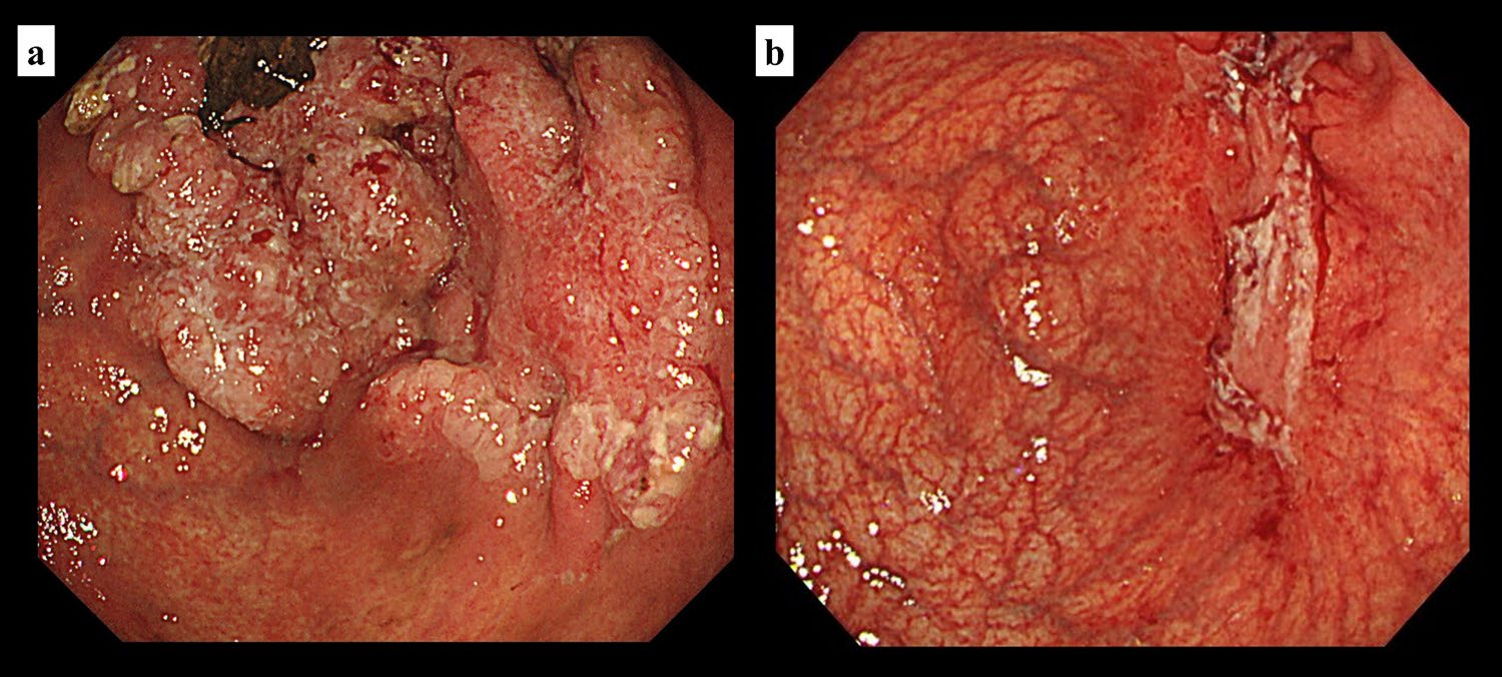
**a** Esophagogastroduodenoscopy before chemotherapy revealed an ulcerative lesion located in the antrum. **b** After the completion of three cycles of SOX + Tmab treatment, the primary lesion decreased significantly in the size, resulting in the formation of a white, lichenoid scar

**Fig. 2:**
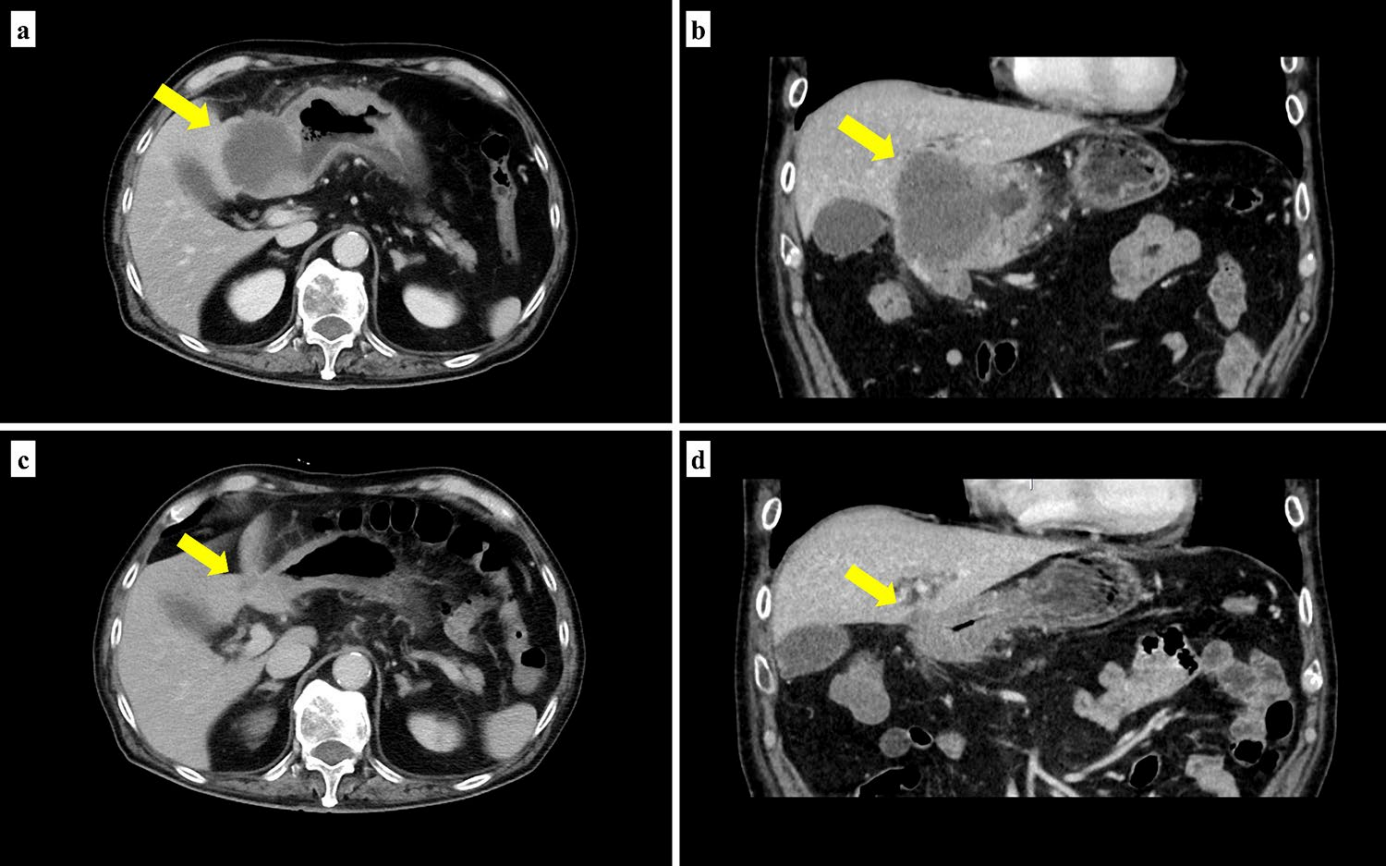
Contrast-enhanced computed tomography. **a**, **b** Before chemotherapy (yellow arrows: primary tumor, hilar lymph nodes, and S4 segment of the liver were involved). **c**, **d** After three cycles of SOX + Tmab

**Fig. 3:**
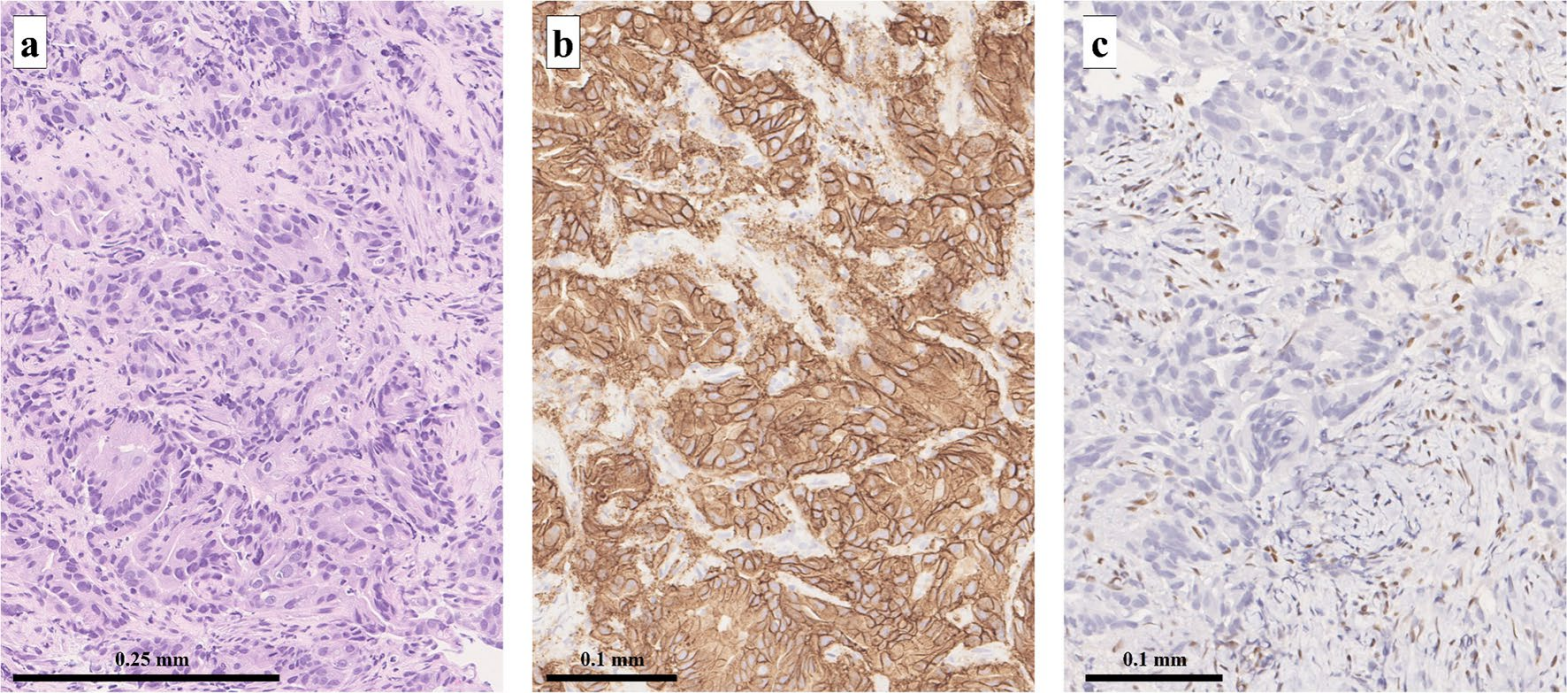
**a** Hematoxylin and eosin-stained section demonstrating features of an intermediate to poorly differentiated adenocarcinoma. **b** HER2 staining intensity was scored at 3 + , indicating overexpression. **c** Negative MLH1 immunostaining, indicating mismatch repair deficiency

A 75-year-old, male patient presented with anorexia and weight loss. Upper gastrointestinal endoscopy revealed an ulcerative lesion in the antrum extending from the lower stomach (Fig. 1a). A biopsy of the lesion led to the diagnosis of adenocarcinoma. Computed tomography revealed that the primary tumor merged with enlarged hilar lymph nodes and infiltrated the S4 segment of the liver, appearing as a low-attenuation area (Fig. 2a, b). Additionally, one enlarged lymph nodes were observed along the lesser curvature of the stomach. These findings led to the diagnosis of stage IVA advanced gastric cancer (T4b(HEP)N1M0) according to the Union for International Cancer Control’s 8th edition criteria[15]. Immunohistochemical examination found HER2 3 + and loss of the MLH1 protein (Fig. 3), and MSI-H was confirmed using the MSI test kit (FALCO). Due to the extensive infiltration into the liver, the treatment plan was to precede with chemotherapy followed by surgery.

**Fig. 4:**
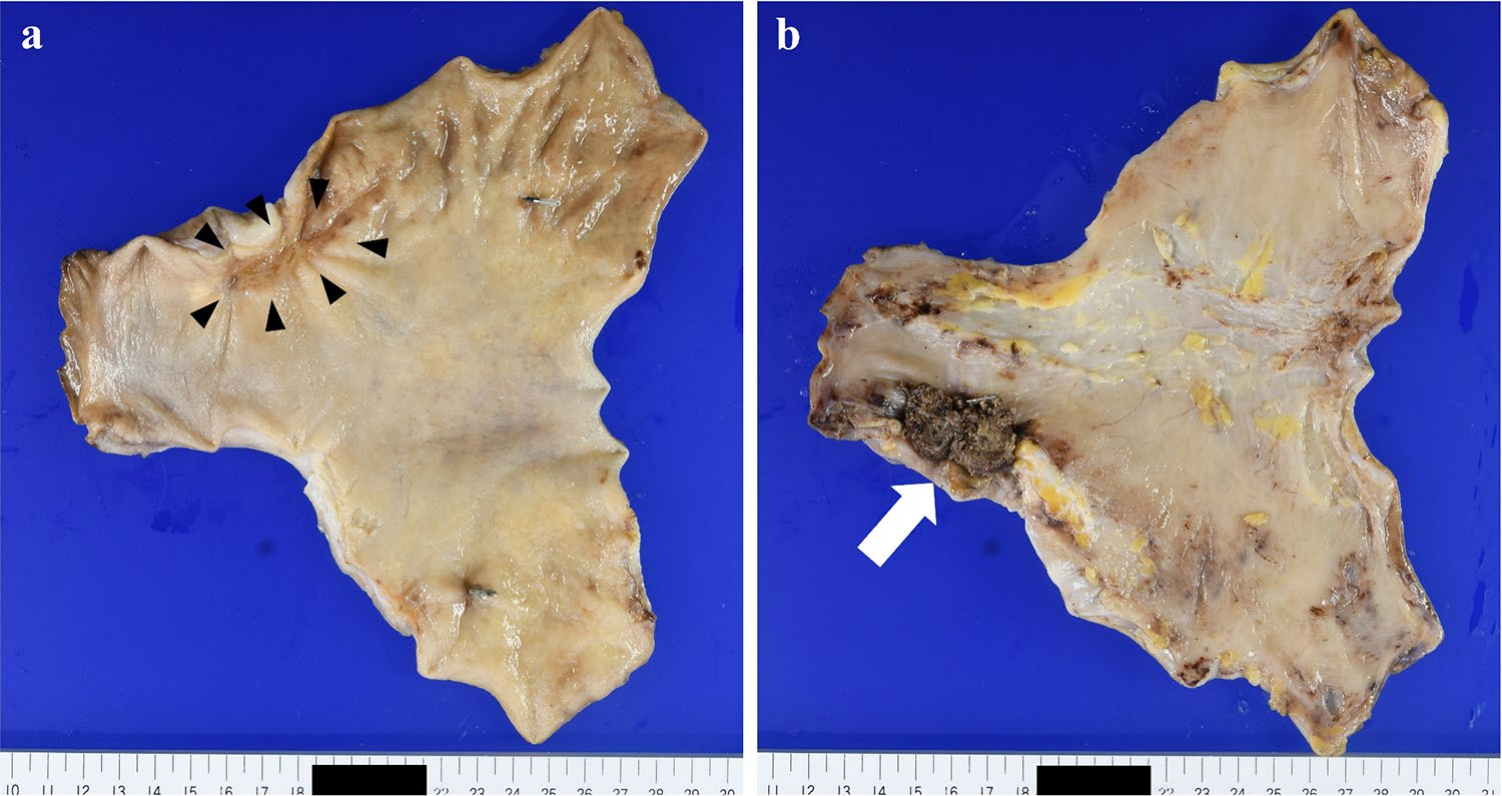
**a** Resected stomach demonstrating a scar from an ulcer at the tumor site (arrowhead). **b** Resected liver mass (30 × 20 × 10 mm) on the serosal surface (white arrow)

**Fig. 5:**
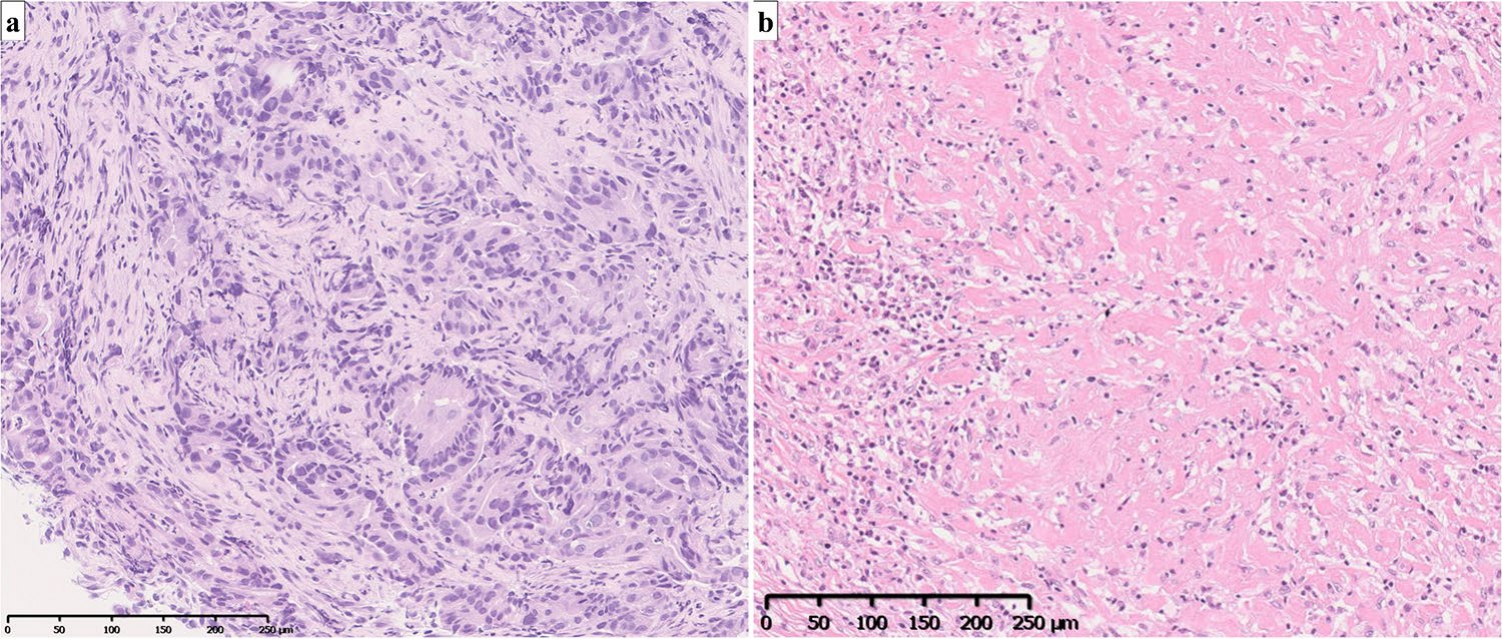
**a** Preoperative biopsy: hematoxylin and eosin-stained sections demonstrate features consistent with intermediate to poorly differentiated adenocarcinoma. **b** Postoperative analysis: examination of the resected gastric tissue reveals no evidence of viable tumor cells

After three cycles of chemotherapy with S-1, oxaliplatin, and trastuzumab (SOX + Tmab), the primary tumor, associated lymph node metastases, and hepatic invasion shrank markedly (Figs. 1b, 2c, d). Serum tumor marker levels remained within normal ranges during the initial diagnosis and chemotherapy. The patient subsequently underwent a gastrectomy and hepatectomy as conversion surgery (Fig. 4), achieving a R0 resection with a pathologically complete response (Fig. 5). A transient elevation in tumor markers was observed postoperatively, followed by a declining trend. The patient resumed SOX + Tmab postoperatively. However, he had prolonged neutropenia and bacteremia, which appeared to be due to mucositis. Following a detailed discussion with the patient regarding the potential benefits and risks, the decision was made to discontinue postoperative chemotherapy. The patient was then placed under observation without any further treatment. At postoperative month 7, the patient was recurrence-free.

## Discussion

The patient in the present study, who had HER2-positive, MSI-H, advanced gastric cancer, responded favorably to a combination of chemotherapy and trastuzumab. MSI-H occurs much less frequently in HER2-positive cases (0.7%) than in gastric cancer in general, as demonstrated by the KEYNOTE-811 trial [11]. The extreme rarity of this condition may be attributable to the characteristics of HER2-positive tumors, which are typically associated with chromosomal instability and a low mutational burden [16, 17]. HER2 is anticipated to remain a crucial therapeutic target in the future. Novel approaches, including new antibody–drug conjugates and bispecific antibodies targeting this molecule, are currently undergoing clinical evaluation and show promise for integration into standard clinical practice (NCT03821233, NCT04276493). The KEYNOTE-811 trial demonstrated the efficacy of trastuzumab and pembrolizumab with chemotherapy in the treatment of HER2-positive gastric cancer [11], but this combination has not been tested against HER2-positive, MSI-H gastric cancer and has not been approved for use in Japan. While both anti-HER2 agents and ICIs have shown promise in their respective indications, direct comparisons of their efficacy in HER2-positive, MSI-H gastric cancers are currently lacking, highlighting an important area for future research. Further investigation is necessary to determine whether anti-HER2 agents or ICIs have a more potent antitumor effect when combined with chemotherapy if only one of these treatments are available.

Conversion surgery confers a significant survival benefit on patients with advanced gastric cancer particularly when preceded by effective chemotherapy and/or a targeted therapy regimen. The success of this approach is influenced by the biological characteristics of the tumor, its response to preoperative therapy, and the ability to achieve a R0 resection [14, 18]. As preoperative therapy, systemic chemotherapy with or without ICIs is effective [12–14, 19, 20]. For patients with HER2-positive gastric cancer, administering chemotherapy with trastuzumab preoperatively has proven to be beneficial [21]. Also, a previous study reported the efficacy of pembrolizumab followed by conversion surgery in a patient with MSI-H [22]. However, whether intensive chemotherapy, chemotherapy with ICIs or anti-HER2 therapy for HER2-positive cases is the optimal pre-conversion surgery regimen, is unclear. It is even more challenging to decide whether to use ICIs, anti-HER2 agents or a combination of these with chemotherapy as preoperative chemotherapy for MSI-H and HER2-positive cancer.

The present patient received a combination of trastuzumab and chemotherapy but did not receive any ICIs. The patient showed a highly favorable response, enabling conversion surgery to be performed, and a pathologically complete response was confirmed. MSI-H gastric cancers typically show reduced sensitivity to fluoropyrimidines [23]. In this case, strong HER2 expression (3+) may have enhanced trastuzumab’s efficacy [3], potentially offsetting the chemoresistance related to MSI-H. However, studies specifically comparing the efficacy of trastuzumab in MSI-H versus MSS gastric cancers are currently lacking. Given the potency of ICI therapy against MSI-H gastric cancer [9, 24, 25], an even better response might have been attained by the administration of additional ICIs. Moreover, the impressive survival outcome achieved in patients with MSI-H gastric cancer using nivolumab and ipilimumab in the CheckMate 649 trial suggested that incorporating ICIs into the chemotherapy regimen may obviate the need for surgical intervention [25].

There is, as of yet, no consensus on the efficacy of additional chemotherapy after conversion surgery. Some studies suggested that continuing chemotherapy postoperatively may confer additional benefits irrespective of HER2 status, and this approach is often used in clinical practice [26, 27]. However, there is no clear evidence of this treatment’s efficacy. Similarly, in the case of HER2-positive gastric cancer, there is no evidence verifying the efficacy of combining postoperative trastuzumab with chemotherapy. Moreover, MSI-H gastric cancer has a better outcome following surgery and benefits less from perioperative chemotherapy [23]. These findings indicate the lack of sufficient evidence and led to the discontinuation of postoperative chemotherapy in the present patient.

In conclusion, the present report is the first to describe the efficacy of systemic chemotherapy with trastuzumab followed by conversion surgery in a patient with HER2-positive, MSI-H, advanced gastric cancer. The present case highlights the need for further research into molecularly targeted treatments and surgical resection in this rare type of cancer.

## Data Availability

The data that support the findings of this study are available from the corresponding author upon reasonable request.

## References

[1] Iqbal N, Iqbal N (2014) Human epidermal growth factor receptor 2 (HER2) in cancers: overexpression and therapeutic implications. Mol Biol Int 2014:852748 25276427 10.1155/2014/852748 PMC4170925

[2] Giuffrè G, Ieni A, Barresi V, Caruso RA, Tuccari G (2012) HER2 status in unusual histological variants of gastric adenocarcinomas. J Clin Pathol 65:237–241 22067088 10.1136/jclinpath-2011-200345

[3] Bang Y-J, Van Cutsem E, Feyereislova A, Chung HC, Shen L, Sawaki A et al (2010) Trastuzumab in combination with chemotherapy versus chemotherapy alone for treatment of HER2-positive advanced gastric or gastro-oesophageal junction cancer (ToGA): a phase 3, open-label, randomised controlled trial. Lancet 376:687–697 20728210 10.1016/S0140-6736(10)61121-X

[4] Shitara K, Bang Y-J, Iwasa S, Sugimoto N, Ryu M-H, Sakai D et al (2020) Trastuzumab deruxtecan in previously treated HER2-positive gastric cancer. N Engl J Med 382:2419–2430 32469182 10.1056/NEJMoa2004413

[5] Janjigian YY, Shitara K, Moehler M, Garrido M, Salman P, Shen L et al (2021) First-line nivolumab plus chemotherapy versus chemotherapy alone for advanced gastric, gastro-oesophageal junction, and oesophageal adenocarcinoma (CheckMate 649): a randomised, open-label, phase 3 trial. Lancet 398:27–40 34102137 10.1016/S0140-6736(21)00797-2 PMC8436782

[6] Kang Y-K, Chen L-T, Ryu M-H, Oh D-Y, Oh SC, Chung HC et al (2022) Nivolumab plus chemotherapy versus placebo plus chemotherapy in patients with HER2-negative, untreated, unresectable advanced or recurrent gastric or gastro-oesophageal junction cancer (ATTRACTION-4): a randomised, multicentre, double-blind, placebo-controlled, phase 3 trial. Lancet Oncol 23:234–247 35030335 10.1016/S1470-2045(21)00692-6

[7] Shitara K, Van Cutsem E, Bang Y-J, Fuchs C, Wyrwicz L, Lee K-W et al (2020) Efficacy and safety of pembrolizumab or pembrolizumab plus chemotherapy vs chemotherapy alone for patients with first-line, advanced gastric cancer: The KEYNOTE-062 phase 3 randomized clinical trial. JAMA Oncol 6:1571–1580 32880601 10.1001/jamaoncol.2020.3370 PMC7489405

[8] Kang Y-K, Boku N, Satoh T, Ryu M-H, Chao Y, Kato K et al (2017) Nivolumab in patients with advanced gastric or gastro-oesophageal junction cancer refractory to, or intolerant of, at least two previous chemotherapy regimens (ONO-4538-12, ATTRACTION-2): a randomised, double-blind, placebo-controlled, phase 3 trial. Lancet 390:2461–2471 28993052 10.1016/S0140-6736(17)31827-5

[9] Chao J, Fuchs CS, Shitara K, Tabernero J, Muro K, Van Cutsem E et al (2021) Assessment of pembrolizumab therapy for the treatment of microsatellite instability-high gastric or gastroesophageal junction cancer among patients in the KEYNOTE-059, KEYNOTE-061, and KEYNOTE-062 clinical trials. JAMA Oncol 7:895–902 33792646 10.1001/jamaoncol.2021.0275 PMC8017478

[10] Akagi K, Oki E, Taniguchi H, Nakatani K, Aoki D, Kuwata T et al (2021) Real-world data on microsatellite instability status in various unresectable or metastatic solid tumors. Cancer Sci 112:1105–1113 33403729 10.1111/cas.14798 PMC7935787

[11] Janjigian YY, Kawazoe A, Yañez P, Li N, Lonardi S, Kolesnik O et al (2021) The KEYNOTE-811 trial of dual PD-1 and HER2 blockade in HER2-positive gastric cancer. Nature 600:727–730 34912120 10.1038/s41586-021-04161-3 PMC8959470

[12] Yoshida K, Yasufuku I, Terashima M, Young Rha S, Moon Bae J, Li G et al (2022) International retrospective cohort study of conversion therapy for stage IV gastric cancer 1 (CONVO-GC-1). Ann Gastroenterol Surg 6:227–240 35261948 10.1002/ags3.12515 PMC8889854

[13] Yamaguchi K, Yoshida K, Tanahashi T, Takahashi T, Matsuhashi N, Tanaka Y et al (2018) The long-term survival of stage IV gastric cancer patients with conversion therapy. Gastric Cancer 21:315–323 28616743 10.1007/s10120-017-0738-1 PMC5846815

[14] Morgagni P, Solaini L, Framarini M, Vittimberga G, Gardini A, Tringali D et al (2018) Conversion surgery for gastric cancer: a cohort study from a western center. Int J Surg 53:360–365 29654967 10.1016/j.ijsu.2018.04.016

[15] Brierley JD, Gospodarowicz MK, Wittekind C (2016) TNM Classification of Malignant Tumours, 8th edn. Wiley, Cham

[16] Castellanos G, Valbuena DS, Pérez E, Villegas VE, Rondón-Lagos M (2023) Chromosomal instability as enabling feature and central hallmark of breast cancer. Breast Cancer 15:189–211 36923397 10.2147/BCTT.S383759 PMC10010144

[17] Janjigian YY, Sanchez-Vega F, Jonsson P, Chatila WK, Hechtman JF, Ku GY et al (2018) Genetic predictors of response to systemic therapy in esophagogastric cancer. Cancer Discov 8:49–58 29122777 10.1158/2159-8290.CD-17-0787 PMC5813492

[18] Sato Y, Ohnuma H, Nobuoka T, Hirakawa M, Sagawa T, Fujikawa K et al (2017) Conversion therapy for inoperable advanced gastric cancer patients by docetaxel, cisplatin, and S-1 (DCS) chemotherapy: a multi-institutional retrospective study. Gastric Cancer 20:517–526 27553665 10.1007/s10120-016-0633-1

[19] Shin M-K, Choi M-G, Kim S-T, Kang W-K, Sohn T-S, An J-Y et al (2023) The clinical implication of conversion surgery in patients with stage IV gastric cancer who received systemic chemotherapy. Biomedicines 11(11):3097. 10.3390/biomedicines11113097 38002099 10.3390/biomedicines11113097 PMC10669208

[20] Hojo Y, Ishida Y, Tomita T, Kurahashi Y, Nakamura T, Kitayama Y et al (2023) Treatment strategy for successful conversion surgery in clinical stage IVB gastric cancer. Eur J Surg Oncol 50:107314 38101115 10.1016/j.ejso.2023.107314

[21] Zhang Y, Xu X, Hu C, Du Y, Ding G, Chen J et al (2022) Trastuzumab in combination with chemotherapy for HER2-positive metastatic gastric cancer patients underwent conversion therapy. Transl Cancer Res 11:2145–2156 35966292 10.21037/tcr-21-2886 PMC9372222

[22] Hidaka Y, Arigami T, Osako Y, Desaki R, Hamanoue M, Takao S et al (2022) Conversion surgery for microsatellite instability-high gastric cancer with a complete pathological response to pembrolizumab: a case report. World J Surg Oncol 20:193 35689267 10.1186/s12957-022-02661-8 PMC9185925

[23] Smyth EC, Wotherspoon A, Peckitt C, Gonzalez D, Hulkki-Wilson S, Eltahir Z et al (2017) Mismatch repair deficiency, microsatellite instability, and survival: an exploratory analysis of the medical research council adjuvant gastric infusional chemotherapy (MAGIC) trial. JAMA Oncol 3:1197–1203 28241187 10.1001/jamaoncol.2016.6762 PMC5824280

[24] Kawakami H, Hironaka S, Esaki T, Chayama K, Tsuda M, Sugimoto N et al (2021) An investigator-initiated phase 2 study of nivolumab plus low-dose Ipilimumab as first-line therapy for microsatellite instability-high advanced gastric or esophagogastric junction cancer (NO LIMIT, WJOG13320G/CA209–7W7). Cancers 13(4):805. 10.3390/cancers13040805 33671871 10.3390/cancers13040805 PMC7918984

[25] Shitara K, Ajani JA, Moehler M, Garrido M, Gallardo C, Shen L et al (2022) Nivolumab plus chemotherapy or ipilimumab in gastro-oesophageal cancer. Nature 603:942–948 35322232 10.1038/s41586-022-04508-4 PMC8967713

[26] Wang T, Wang N, Ren H, Zhou H, Zhou A, Jin J et al (2019) Long-term results of conversion therapy for initially unresectable gastric cancer: analysis of 122 patients at the national cancer center in China. J Cancer 10:5975–5985 31762807 10.7150/jca.35527 PMC6856572

[27] Nakanishi K, Tanaka C, Kanda M, Miyata K, Furukawa K, Maeda O et al (2023) Low expectancy of conversion surgery with r0 resection in patients with CEA > 5.0 ng/mL at the initial recist evaluation for metastatic gastric cancer. Cancers 15(21):5197. 10.3390/cancers15215197 37958371 10.3390/cancers15215197 PMC10650046

